# Methylenetetrahydrofolate Reductase 677T Allele Is a Risk Factor for Arterial Thrombosis in Chinese Han Patients with Antiphospholipid Syndrome

**DOI:** 10.3390/biomedicines11010055

**Published:** 2022-12-26

**Authors:** Zihan Tang, Hui Shi, Honglei Liu, Xiaobing Cheng, Yutong Su, Junna Ye, Yue Sun, Qiongyi Hu, Huihui Chi, Zhuochao Zhou, Jinchao Jia, Jianfen Meng, Mengyan Wang, Fan Wang, Jialin Teng, Chengde Yang, Tingting Liu

**Affiliations:** Department of Rheumatology and Immunology, Ruijin Hospital, Shanghai Jiao Tong University School of Medicine, Shanghai 200025, China

**Keywords:** antiphospholipid syndrome, arterial thrombosis, methylenetetrahydrofolate reductase C677T polymorphism

## Abstract

Antiphospholipid syndrome (APS) is a systemic autoimmune disorder characterized by the persistent presence of antiphospholipid antibodies (aPL) and thrombotic or obstetric events. Given the heterogeneity of the clinical manifestations, it is likely that genetic and acquired factors are involved in the pathogenesis of APS. The inherited polymorphisms of the thrombophilic gene, including methylenetetrahydrofolate reductase (MTHFR) C677T, type 1 plasminogen activator inhibitor (PAI-1) 4G/5G, factor V Leiden (FVL) G1691A, prothrombin (PT) G20210A, antithrombin (AT), and fibrinogen (Fg) polymorphisms, were analyzed in 67 aPL(+) patients from the Chinese Han population, including 41 APS patients and 26 persistent aPL carriers. The MTHFR C677T genotypes of 105 healthy controls, and the PAI-1 4G/5G polymorphism of 120 healthy controls, from the Chinese Han population were acquired for this study. Both the MTHFR C677T genotype (χ^2^ = 10.67, *p* = 0.004) and C/T allele distribution (χ^2^ = 5.92, *p* = 0.019) between the aPL(+) patients and healthy controls were found to be significantly different. Furthermore, we observed that the patients with at least one T allele had a higher risk of arterial thrombosis (CT vs. CC, OR 11.00, *p*= 0.025; CT + TT vs. CC, OR 10.27, *p* = 0.018). The C677T mutation of MTHFR is a risk factor for arterial thrombosis in Chinese Han patients with APS.

## 1. Introduction

Antiphospholipid syndrome (APS) is a systemic autoimmune disorder characterized by vascular thrombosis and/or pregnancy morbidity; it occurs in patients with persistent antiphospholipid antibodies (aPLs). APLs are a heterogeneous group of antibodies, including the lupus anticoagulant (LA), anticardiolipin antibody (aCL), and anti-β2 glycoprotein 1 antibody (anti-β2GP1) [1]. The principal manifestations are venous, arterial, or microvascular thrombosis. Obstetric complications may also occur, such as recurrent early miscarriages, fetal loss, or premature births [2,3]. Thrombosis, which commonly presents as a stroke, transient ischemic attacks, deep vein thrombosis, or a pulmonary embolism, are the most frequent clinical manifestations of APS, and they have the potential to cause disability, they have a tendency to recur, and they have a high mortality rate [4]. 

The activation of target cells by aPLs is widely considered to be the driving force for pathologic outcomes in APS; however, not all patients with persistent aPLs develop thrombosis. The heterogeneity of the clinical manifestations associated with APS indicates that aPLs are necessary, but insufficient for the development of APS, and other acquired and/or inherited factors play a role in pushing the hemostatic balance in favor of thrombosis [5]. The accumulating amount of evidence strongly suggests that genetic predisposition plays a critical role in the development of APS. Familial studies support the gene polymorphisms that are related to HLA antigens. Next-generation sequencing identified rare genomic variants (such as IRF5 encoding interferon regulatory factor 5, STAT4 encoding signal, and activator of transcription 4) that may also play a role in APS pathogenesis [6,7,8,9]. It is well established that the inherited thrombophilic gene polymorphisms were associated with aPL- as well as non-aPL-associated thrombosis. The thrombophilic gene polymorphisms, including factor V Leiden, prothrombin G20210A, antithrombin, fibrinogen, type 1 plasminogen activator inhibitor (PAI-1) 4G/5G, and the methylenetetrahydrofolate reductase (MTHFR) C677T polymorphism, are reported to be the genetic determinants of thrombophilia [10,11,12]. Gene polymorphism in factor V Leiden, prothrombin G20210A, and PAI-1 4G/5G were also found to be a thrombotic risk in APS patients [6,13,14]. Some studies concerning both antithrombin and fibrinogen gene polymorphisms in APS have been carried out. Some researchers investigated the antithrombin III (AT III) or α-fibrinogen (FGA) Thr321Ala polymorphism in APS patients, but no association with thrombosis was found [15,16]. Although several studies have suggested that patients with homozygous MTHFR C677T were more likely to be subject to an increased number of thrombosis events, and that they had a lower mean age at the first thrombosis event than patients with non-homozygous MTHFR C677T, previous studies showed no significant difference between the MTHFR C677T genetic distribution in APS patients and the healthy group [13,17,18]. In addition, mutations in the factor V Leiden and prothrombin approximately triple the risk of late fetal loss, and the combination of an antithrombin gene mutation with other thrombophilic mutations result in an increased risk of recurrent pregnancy loss (RPL) [19,20].

Our study aimed to investigate the prevalence of the following important inherited thrombophilia gene polymorphisms: MTHFR C677T, PAI-1 4G/5G, factor V Leiden G1691A, prothrombin G20210A, antithrombin (T7747C, C10446T, 18390-1 ins CT), and fibrinogen (FGA-α G1233A, FGB-β A9692G, FGG-γ G9135T, FGG-γ G10819A, FGG-γ G12688A), in patients with persistent aPLs from the Chinese Han population. We also analyzed the potential relationship between the genetic variants in the thrombophilia-related genes and the clinical manifestations in APS. Our results demonstrated that the C677T mutation of MTHFR is a risk factor of arterial thrombosis in Chinese Han patients with APS.

## 2. Materials and Methods

### 2.1. Patients

**Figure 1:**
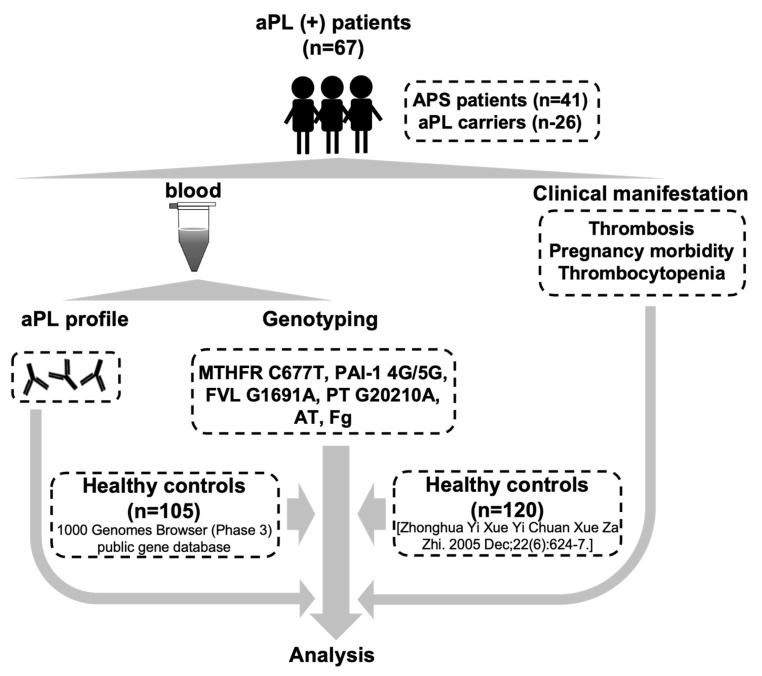
The illustrative figure for the summary of this study.

This study was approved by the Institutional Review Broad of Ruijin Hospital (ID: 2016-61), Shanghai Jiao Tong University School of Medicine, Shanghai, China. Informed consent was obtained from each participant. We enrolled 67 consecutive patients, each with persistent positive antiphospholipid antibodies (aPL(+) patients), from the APS-SH database (APS-Shanghai) which was established by expert rheumatologists and statisticians in 2000 [21]. Of the 67 aPL(+) patients, 41 patients fulfilling the Sydney criteria were diagnosed as having APS (13 male, 28 female; mean age, 40 ± 12 years), and 26 asymptomatic carriers had persistent antiphospholipid antibodies (2 male, 24 female; mean age, 37 ± 12 years). Demographic characteristics of the enrolled aPL(+) patients are summarized in Table 1. Blood was drawn into blood collection tubes containing ethylenediamine tetraacetic acid (EDTA) (which acted as an anticoagulant) by an experienced phlebotomist, and the blood was frozen at −80 °C until testing. The illustrative figure for the summary of this study is shown in Figure 1.

The 1000 Genomes Project created the largest public catalog of human variations and genotype data. In this study, the MTHFR C677T genotypes of 105 healthy controls, who were from the Southern Han Chinese population, were acquired from the 1000 Genomes Browser (Phase 3), which is a public gene database [22]. The distribution of the PAI-1 4G/5G polymorphism in 120 healthy controls in Chinese Han population was obtained from the study of Chen et al. [23].

### 2.2. Genotype Analysis

Genomic DNA was extracted from whole blood. For detection of the MTHFR C677T polymorphism, a described protocol was used, including a polymerase chain reaction, digestion with Hinfl, gel electrophoresis, and restriction fragment length polymorphism analysis [24]. DNA was assayed for the PAI-1 4G/5G polymorphism, using a protocol based on a PCR technique and digestion aided by the Bsi YI restriction enzyme [16]. To detect the gene polymorphisms in factor V Leiden (G1691A), prothrombin (G20210A), antithrombin (T7747C, C10446T, 18390-1 ins CT), and fibrinogen (FGA-α G1233A, FGB-β A9692G, FGG-γ G9135T, FGG-γ G10819A, FGG-γ G12688A), we used allele-specific PCR as previously described [25,26].

### 2.3. Statistical Analysis

All statistical analyses were performed using SPSS version 26.0 (IBM, Chicago, IL, USA). Differences between groups were analyzed using the t-test, Mann–Whitney U-test, χ^2^ test, or Fisher’s exact test, as appropriate. The Hardy–Weinberg equilibrium of the entire cohort was tested using the χ^2^ test [27]. Odds ratios (OR) were determined by logistical regression and the 95% confidence intervals (95% CI) were calculated. *p* values < 0.05 were considered statistically significant.

## 3. Results

Among all the detected inherited thrombophilia gene polymorphisms, including MTHFR C677T, PAI-1 4G/5G, factor V Leiden G1691A, prothrombin G20210A, antithrombin, and the fibrinogen polymorphisms, only the gene mutations in MTHFR C677T and PAI-1 4G/5G were found in the enrolled 67 aPL(+) patients. We compared the genotype distribution of the two genes between the aPL(+) patients and healthy controls, and we found that there was no significant difference between the two groups with regard to PAI-1 4G/5G genotype distribution (χ^2^ = 2.49, *p* = 0.290).

The distribution of the MTHFR C677T genotype and C/T allele in the aPL(+) patients and healthy controls is shown in Table 2. The chi-square test was used to compare the MTHFR C677T genotype and C/T allele distribution between the two groups. The distribution of the MTHFR C677T genotypes CC, CT, and TT were 26.8%, 62.7%, and 10.5% in the aPL(+) patients, versus 51.5%, 39.0%, and 9.5% in the healthy controls. The C and T allele frequencies were 58.2% and 41.8% in the aPL(+) patients, and 70.9% and 29.1% in healthy controls, respectively. Both the MTHFR C677T genotype (χ^2^ = 10.67, *p* = 0.004) and C/T allele distribution (χ^2^ = 5.92, *p* = 0.019) between the aPL(+) patients and healthy controls were significantly different. The CT genotype frequency was higher in the aPL(+) patients (OR 3.07, 95% CI 1.55–6.09, *p* = 0.002) than the healthy controls, whereas the TT genotype frequency showed no significant difference between the two groups. Compared with the healthy controls, the aPL(+) patients had a higher susceptibility to the T allele (OR 1.75, 95% CI 1.11–2.76, *p* = 0.019) (Table 2). There was no significant difference between the MTHFR C677T genotype distribution in the APS patients and the aPL(+) carriers (χ^2^ = 0.78, *p* = 0.695). The MTHFR C677T genotype distribution and allele frequencies in both the aPL(+) patients and healthy controls were in accordance with those predicted by the Hardy–Weinberg equilibrium, shown in Table 3 (*p* > 0.05).

The distribution of the MTHFR C677T genotype and the C/T allele in APS patients, according to their clinical manifestations, is shown in Table 4. There were no differences with regard to the MTHFR C677T genotype or C/T allele distribution when we compared APS patients with thrombosis or pregnancy morbidity, to those without thrombosis or pregnancy morbidity. Thrombosis events in APS patients involve veins, arteries, and microvasculature. This variability in terms of the location of the thrombi results in a wide spectrum of clinical presentations. Moreover, there was no statistically significant difference between subgroups with or without arterial thrombosis, venous thrombosis, early pregnancy morbidity, or late pregnancy morbidity; although, a trend for the CT genotype frequency to be higher in APS patients with arterial thrombosis became apparent, which was absent in patients without arterial thrombosis. Unexpectedly, we found that the distribution of the MTHFR C677T genotype was significantly different between APS patients with and without thrombocytopenia (*p* = 0.040), although the frequency of the C/T allele was 0.37/0.63 in APS patients with thrombocytopenia; this was not significantly different from its frequency (0.64/0.36) in patients without thrombocytopenia (*p* = 0.088).

The risk of arterial thrombosis in APS patients, which is related to the MTHFR C677T genotype and C/T allele, was analyzed using logistic regression, and the results are shown in Table 5. The frequency of MTHFR C677T genotype in patients with arterial thrombosis were 6.7% for CC, 80.0% for CT, and 13.3% for TT. In patients without arterial thrombosis, 42.3% of the subjects had the CC genotype, 46.2% had the CT genotype and 11.5% had the TT genotype. The OR for arterial thrombosis in APS patients with the CT genotype was 11.00, compared with patients who had the CC genotype (95% CI 1.22–99.07, *p* = 0.025). The APS patients with at least one T allele had a higher risk of arterial thrombosis than patients with the CC allele (OR 10.27, 95% CI 1.17–90.17, *p* = 0.018) (Table 5).

No association between the aPL risk profile and distribution of the MTHFR C677T genotype (χ^2^ = 5.28, *p* = 0.072) or C/T allele (χ^2^ = 1.54, *p* = 0.281) was found in our aPL(+) patients from the Chinese Han population.

## 4. Discussion

APS is a heterogeneous systemic autoimmune disorder in which the persistent presence of aPLs is related to the increased risk of thrombotic or obstetric events [2]. In addition to the criteria for aPL (LA, aCL, and anti-β2GP1), studying the non-criteria of aPL in APS patients showed their role in the pathophysiology of APS [28,29,30,31]; however, not all patients with persistent aPLs develop APS, thus suggesting that other additional factors may contribute to the pathogenesis of APS. In recent decades, great advances in the pathogenesis of thrombosis have been made. Currently, it is well established that both acquired and inherited factors contribute to the thrombophilic state that is associated with aPL- or non-aPL-related thrombosis [5,32].

Gene polymorphism in factor V Leiden, prothrombin G20210A, and PAI-1 4G/5G were also found to be a thrombotic risk in APS patients [6,15,16]. Some studies concerning both antithrombin and fibrinogen gene polymorphisms in APS were carried out. Some researchers investigated the AT III or FGA Thr321Ala polymorphism in APS patients, but no association with thrombosis was found.

We assessed the inherited thrombophilia gene polymorphisms, including MTHFR C677T, PAI-1 4G/5G, factor V Leiden G1691A, prothrombin G20210A, antithrombin (T7747C, C10446T, 18390-1 ins CT), and fibrinogen (FGA-α G1233A, FGB-β A9692G, FGG-γ G9135T, FGG-γ G10819A, FGG-γ G12688A), in patients with aPLs from the Chinese Han population; however, we only found mutations in the MTHFR C677T genotype and PAI-1 4G/5G in our aPL(+) patients. A previous study showed that the occurrence of factor V Leiden and the prothrombin G20210A variant were 1% and 6% in APS patients, respectively [7]. Moreover, the AT III and FGA Thr321Ala polymorphism were also reported in APS patients. As they have been studied in different populations, and ethnicity is a crucial variable to account for with regard to interindividual variability, we suggest that further elucidation of these results, with a much larger sample size, may help in better understanding the roles of factor V Leiden, prothrombin, antithrombin, and fibrinogen gene variants in APS.

The enzyme MTHFR catalyzes the irreversible conversion of 5,10-methylenetetrahydrofolate to 5-methyltetrahydrofolate, which is the methyl donor for the re-methylation of homocysteine to methionine [33]. The MTHFR locus is mapped onto chromosome 1 (1p36.3) and a common alternation in the MTHFR gene, C677T, converts an alanine to a valine at codon 222, causing decreased activity in the enzyme [25]. The inability of the MTHFR enzyme leads to a rise in plasma homocysteine levels. Moreover, the homozygous mutation has higher homocysteine levels, whereas the heterozygous mutation has a mild increase in homocysteine levels compared with the non-mutated controls [34]. The MTHFR C677T polymorphism is associated with various diseases, such as vascular diseases, infertility, neurological diseases, and cancers [6].

Several studies focused on the association between the MTHFR C677T polymorphism and antiphospholipid syndrome. The varied results may partially be attributed to different sample sizes and population groups. Galli et al. found that the MTHFR C677T mutation was not a risk factor for venous or arterial thrombosis in patients with lupus anticoagulants [35]. A study including 22 women with APS and 41 healthy fertile controls found that the MTHFR C677T mutation and hyperhomocysteinemia were not common in women with APS [36]. Another study also found that the MTHFR C677T polymorphism was equally distributed among the four groups, including the thrombotic group without APS, healthy controls, APS, and aPL(+) patient groups; however, the APS patients who had a homozygous genotype for the MTHFR C677T polymorphism had a lower age at their first thrombosis event, they had more thrombotic events, and higher homocysteine levels, compared with those APS patients who had a non-homozygous genotype for polymorphisms [15].

Opposing the findings of previous studies, unexpectedly, we found that the distribution of the MTHFR C677T polymorphism in our 67 aPL(+) patients was significantly different from the distribution in the 105 healthy controls; these patients were enrolled from the 1000 Genomes Browser (Phase 3) public gene database [22]. Both the enrolled aPL(+) patients and healthy controls were from the Han Chinese population; however, the MTHFR C677T polymorphisms were similarly distributed between the APS patients and aPL carriers. It is the first time that we reported on the distribution of the MTHFR C677T polymorphisms in a cohort of aPL(+) patients from the Chinese Han population, including 41 defined APS patients and 26 persistent aPL carriers.

When we analyzed the MTHFR C677T genotypic distribution and C/T allele frequencies in the subgroups of the 41 APS patients, in accordance with their clinical manifestations, we failed to correlate the MTHFR C677T polymorphism with thrombosis or pregnancy morbidity. We also did not find a correlation in the subgroup wherein we analyzed arterial/ venous thrombosis and early/late pregnancy morbidity in APS patients. In addition, we analyzed the risk of clinical manifestations in APS that is related to the MTHFR C677T genotype and C/T allele. Importantly, the patients with at least one T allele had a higher risk of arterial thrombosis. Thrombocytopenia is a well-recognized feature of APS. The pathogenesis of thrombocytopenia in APS has not been clearly demonstrated. Patients with aPLs and hemocytopenia, who had no history of thrombosis or pregnancy morbidity, are not classified as APS; however, this combination of findings may indeed represent a prethrombotic state that precedes the onset of APS. To our surprise, we found that the distribution of the MTHFR C677T genotype in APS patients, with or without thrombocytopenia, was significantly different (*p* = 0.040). Our study first unraveled that the MTHFR C677T mutation is a risk factor for arterial thrombosis in Chinese Han patients with APS.

Although there was no significant difference in the MTHFR C677T genotype distribution between the APS patients and the aPL(+) carriers, the performance of the MTHFR 677T polymorphism in the current study provides researchers with guidelines for the future. A prospective study with a larger sample size could help elucidate the longitudinal relationship between the MTHFR 677T allele and the development of arterial thrombosis in aPL carriers. With respect to our study limitations, the sample size of aPL(+) patients included in the present study was relatively small. Moreover, we only enrolled members of the Chinese Han population in this study. As ethnicity is a crucial variable to account for with regard to interindividual variability, studies with larger sample size and various ethnic groups may help to elucidate the relationship between the inherited gene polymorphisms and the pathogenesis of aPL-related thrombosis.

We also examined PAI-1 4G/5G polymorphisms in our aPL(+) patients. PAI-1 is a serine protease inhibitor, regulating fibrinolysis and thrombosis via the inhibiting tissue plasminogen activator (tPA) and urokinase (uPA) [37]. Several studies reported that PAI-1 4G polymorphism may influence the expression of PAI-1, and it should be taken into consideration as a risk factor in patients with thrombosis [16,38,39]. In accordance with the result of a previous study performed by Tassies et al. [16], our patients did not have a higher risk of the PAI-1 4G mutation when we compared their genotypic distribution with the healthy controls (*p* = 0.443); however, our results were different to the results from the study performed by Tassies et al. [16] as they reported that the presence of the 4G allele of the 4G/5G polymorphism in the PAI-1 gene is a risk factor for the development of arterial thrombosis in APS patients. The PAI-1 4G/5G mutation did not increase the risk for the development of thrombosis, pregnancy morbidity or thrombocytopenia in our APS patients, which may be due to the genetic variation between races.

## 5. Conclusions

In conclusion, the presence of the MTHFR 677T allele is a risk factor for aPL(+) patients and the potential development of arterial thrombosis in the Chinese Han population.

## Figures and Tables

**Table 1:** Baseline characteristics of the enrolled aPL(+) patients.

	APS	aPL Carrier	*p* Value
n	41	26	
Sex (M/F)	13/28	2/24	0.023
Age (median(q1–q3))	38 (32–47)	34 (30–42)	0.221
Clinical manifestations, n (%)			
Thrombosis	33 (80.5)	/	/
AT only	10 (24.4)	/	/
VT only	18 (43.9)	/	/
AT + VT	5 (12.2)	/	/
Pregnancy morbidity	12 (29.3)	/	/
EPM	2 (4.9)	/	/
LPM	9 (21.9)	/	/
EPM + LPM	1 (2.4)	/	/
Thrombocytopenia	8 (19.5)	4 (15.4)	0.933
aPL profile, n (%)			
Positive aCL IgG	19 (46.3)	15 (57.7)	0.573
Positive aCL IgM	10 (24.4)	12 (46.2)	0.002
Positive anti-β2GP1 IgG	12 (29.3)	7 (26.9)	0.531
Positive anti-β2GP1 IgM	6 (14.6)	4 (15.4)	0.206
Positive LAC	31 (75.6)	16 (61.5)	0.113
Low-risk aPL ^†^	6 (14.6)	2 (7.7)	0.469
High-risk aPL ^‡^	35 (85.4)	24 (92.3)	0.469

^†^ A low-risk aPL profile is defined as isolated aCL or anti-β2 glycoprotein 1 antibodies at low-medium titers. ^‡^ A high-risk aPL profile is defined as a positive LA test, a double or triple aPL positivity, or if persistently high aPL titers are present. AT, arterial thrombosis; VT, venous thrombosis; EPM, early pregnancy morbidity; LPM, late pregnancy morbidity. /, none.

**Table 2:** MTHFR C677T genotype and allele distribution in aPL(+) patients and healthy controls.

Polymorphism		aPL(+) Patients	Healthy Controls	OR (95% CI)	*p* Value	χ^2^	*p* Value
Genotype, n (%)	CC	18 (26.8)	54 (51.5)	1.00 (referent)		10.67	0.004 *
	CT	42 (62.7)	41 (39.0)	3.07 (1.55–6.09)	0.002 *		
	TT	7 (10.5)	10 (9.5)	2.10 (0.69–6.33)	0.232		
Allele, n (%)	C	78 (58.2)	149 (70.9)	1.00 (referent)			
	T	56 (41.8)	61 (29.1)	1.75 (1.11–2.76)		5.92	0.019 *

* *p* < 0.05.

**Table 3:** MTHFR C667T loci of the Hardy–Weinberg genetic equilibrium test.

Group	Genotype			Allele		χ^2^	*p* Value
	CC Case (%)	CT Case (%)	TT Case (%)	C Case (%)	T Case (%)		
Control	54 (51.5)	41 (39.0)	10 (9.5)	149 (70.9)	61 (29.1)	0.192	0.959
aPL(+) Patients	18 (26.8)	42 (62.7)	7 (10.5)	78 (58.2)	56 (41.8)	2.796	0.261

**Table 4:** MTHFR C677T genotype and clinical manifestations in APS patients.

	Genotype, CC, CT, TT, No. of Patients	*p* Value	Allele Frequency C/T	*p* Value
Criteria manifestation				
Thrombosis				
Yes (n = 33)	8, 21, 4	0.577	0.56/0.44	0.409
No (n = 8)	4, 3, 1		0.69/0.31	
Arterial thrombosis				
Yes (n = 15)	1, 12, 2	0.052	0.47/0.53	0.110
No (n = 26)	11,12, 3		0.65/0.35	
Venous thrombosis				
Yes (n = 23)	8, 12, 3	0.737	0.61/0.39	0.658
No (n = 18)	4, 12, 2		0.56/0.44	
Pregnancy morbidity				
Yes (n = 12)	5, 5, 2	0.418	0.63/0.37	0.806
No (n = 29)	7, 19, 3		0.57/0.43	
Early pregnancy morbidity				
Yes (n = 3)	1, 2, 0	0.990	0.67/0.33	0.990
No (n = 38)	11, 22, 5		0.58/0.42	
Late pregnancy morbidity				
Yes (n = 10)	4, 4, 2	0.447	0.60/0.40	0.990
No (n = 31)	8, 20, 3		0.58/0.42	
Non-criteria manifestation				
Thrombocytopenia				
Yes (n = 8)	0, 6, 2	0.040 *	0.37/0.63	0.088
No (n = 33)	12, 18, 3		0.64/0.36	

* *p* < 0.05.

**Table 5:** Arterial thrombosis and MTHFR C677T polymorphism in APS patients.

Polymorphism	Arterial Thrombosis, n (%)	No Arterial Thrombosis, n (%)	OR (95% CI)	*p* Value
Genotype				
CC	1 (6.7)	11 (42.3)	1.00 (referent)	
CT	12 (80.0)	12 (46.2)	11.00 (1.22–99.07)	0.025 *
TT	2 (13.3)	3 (11.5)	7.33 (0.484–111.19)	0.191
CT + TT	14 (93.3)	15 (57.7)	10.27 (1.17–90.17)	0.018 *
Allele				
C	14 (46.7)	34 (65.4)	1.00 (referent)	
T	16 (53.3)	18 (34.6)	2.16 (0.86–5.40)	0.110

* *p* < 0.05.

## Data Availability

All the data are included in the article or available from the authors for reasonable request.
